# Treatment of large gastric trichobezoar in children: Two case reports and literature review

**DOI:** 10.1097/MD.0000000000033589

**Published:** 2023-04-21

**Authors:** Hyung Jun Kwon, Jinyoung Park

**Affiliations:** a Department of Surgery, School of Medicine, Kyungpook National University, Kyungpook National University Hospital, Daegu, South Korea.

**Keywords:** case report, children, trichobezoar, trichophagia, trichotillomania

## Abstract

Gastric trichobezoars are a rare form of bezoar formed from swallowed human hair as well as hair from dolls or animals, blankets, and carpets. They usually develop in young women who are emotionally disturbed, depressed, or mentally retarded, with trichotillomania and trichophagia. They can lead to abdominal pain, gastric ulceration, bleeding, obstruction, and perforation.

**Patient concerns:**

A 13-year-old girl was admitted to our institution with complaints of nausea, vomiting, and epigastric pain for 2 days. She underwent laparoscopic removal of a large gastric trichobezoar at our institution 5 years ago. A 12-year-old girl presented at our institution due to epigastric pain, dizziness, and melena for 2 days.

**Diagnosis:**

They were diagnosed with gastric trichobezoar by abdominal computed tomography scan and upper gastrointestinal endoscopy.

**Interventions:**

Large gastric trichobezoars were removed via a mini-laparotomy.

**Outcomes:**

They recovered well postoperatively without complication.

**Lessons:**

Although the recurrence of gastric trichobezoar after surgery is rare, few recurrent cases were reported in the literature. Therefore, psychiatric consultation and regular follow-up after treatment should be considered in the children and their parents to prevent the recurrence of gastric trichobezoar.

## 1. Introduction

Gastric trichobezoars are a rare form of bezoar formed from swallowed human hair as well as hair from dolls or animals, blankets, and carpets.^[1–4]^ They usually develop in young women who are emotionally disturbed, depressed, or mentally retarded, with trichotillomania and trichophagia.^[5–9]^ They can lead to abdominal pain, gastric ulceration, bleeding, obstruction, and perforation.^[6–8]^ Psychiatric consultation and regular follow-up after treatment are important to prevent the recurrence of trichobezoar. We report 2 cases of a large gastric trichobezoar in children with trichotillomania and trichophagia that were successfully treated surgically.

## 2. Case report

### 2.1. Case 1

**Figure 1.**
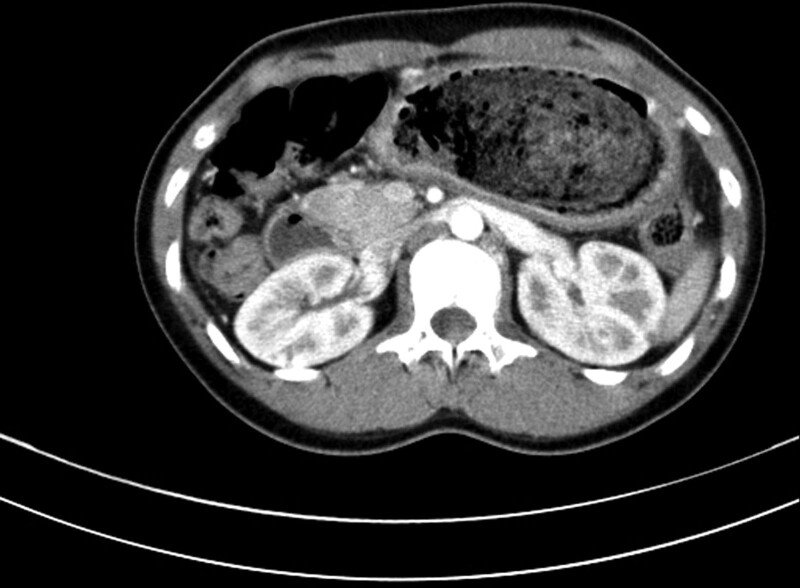
Axial view of abdominal CT scan demonstrates a large intraluminal mass with entrapped air obstructing entire lumen of the stomach. CT = computed tomography.

**Figure 2.**
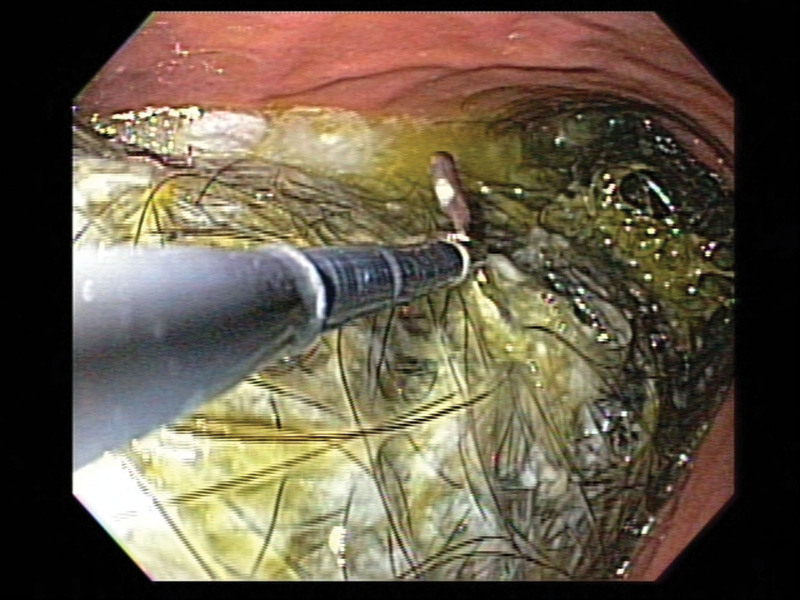
Recurrent gastric trichobezoar is confirmed by upper gastrointestinal endoscopy.

A 13-year-old girl was admitted to our institution with complaints of nausea, vomiting, and epigastric pain for 2 days. She had a previous history of trichotillomania and trichophagia. She underwent laparoscopic removal of a large gastric trichobezoar at our institution 5 years ago. Psychiatric evaluation and counseling have been done after the 1st surgery. However, she has been lost to follow-up. On abdominal physical examination at the second visit, a large, hard mobile nontender mass was palpated in the epigastric area. There was a bald area on her scalp. Abdominal computed tomography (CT) scan demonstrated a large intraluminal mass obstructing the entire lumen of the stomach (Fig. 1). The mass was confirmed to be a gastric trichobezoar by upper gastrointestinal endoscopy (Fig. 2). An endoscopic attempt to fragment and remove the gastric trichobezoar was tried 3 times under sedation. However, it failed because the gastric trichobezoar was too large and badly tangled. Laparoscopic removal of gastric trichobezoar was performed. Totally laparoscopic removal was not possible due to the large size of the gastric trichobezoar. Therefore, the left paraumbilical port incision was extended and the stomach was delivered through the extended incision. A 15 × 6 cm trichobezoar was retrieved through a longitudinal gastrotomy over the gastric antrum, which was similar to that of the previous gastric trichobezoar removed 5 years ago. She recovered well postoperatively without complication. She and her parent were referred to a pediatric psychiatrist again to prevent the recurrence.

### 2.2. Case 2

**Figure 3.**
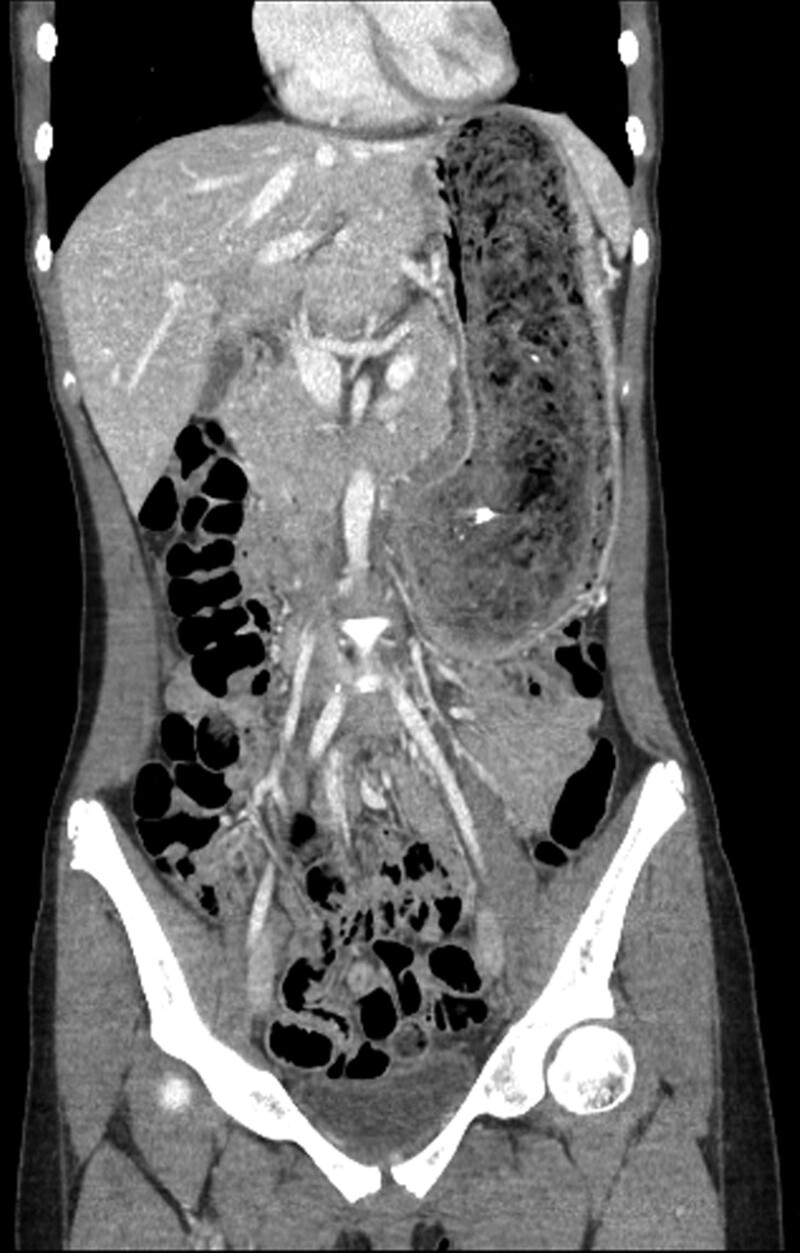
Coronal view of abdominal CT scan demonstrates a large intraluminal mass occupying the entire stomach. CT = computed tomography.

**Figure 4.**
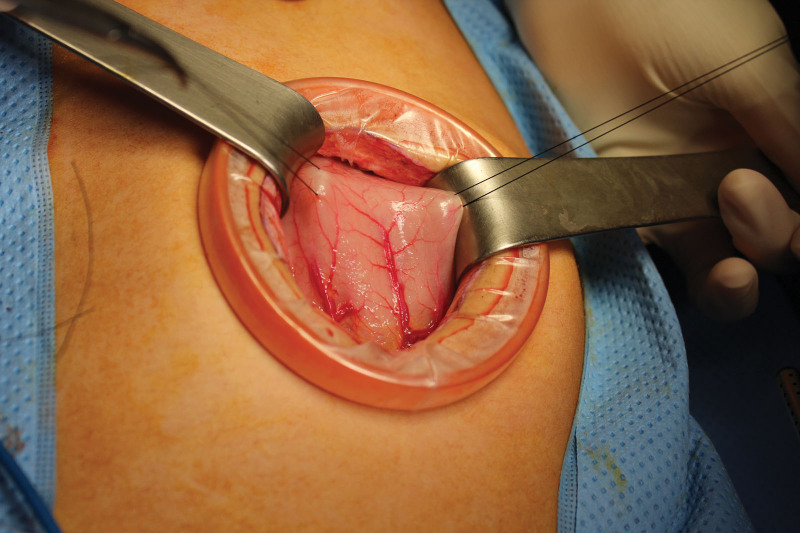
Wound retractor is used to expose the anterior wall of the stomach.

**Figure 5.**
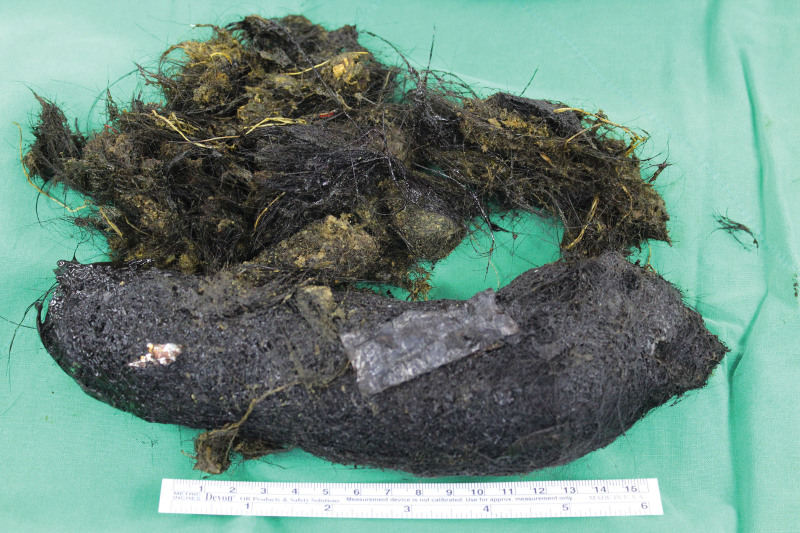
A 22 × 7 cm-sized huge gastric trichobezoar after removal.

A 12-year-old girl presented at our institution due to epigastric pain, dizziness, and melena for 2 days. Her parents perceived that she started pulling her hair out and eating them after being stressed out with her studies a year ago. An abdominal CT scan demonstrated a large intraluminal mass occupying the entire stomach (Fig. 3). The decision was made to proceed with open laparotomy instead of laparoscopy due to technical difficulty because the size of the gastric trichobezoar was too large. An exploratory mini-laparotomy around the umbilicus (5 cm) was performed. Wound retractor (Endo Keeper, Nelis Corporation, South Korea) was used to expose the anterior wall of the stomach. The anterior wall of the gastric antrum was brought out onto the anterior abdominal wall and then was opened after 2 interrupted stay silk sutures (Fig. 4). A 22 × 7 cm huge gastric trichobezoar was retrieved without complications (Fig. 5). The gastrotomy was primarily repaired with a nonabsorbable suture. Her postoperative course was uneventful. She and her parent has been under pediatric psychiatric evaluation and treatment. She has done well at a follow-up of 4 years.

## 3. Discussion

Bezoars typically develop in the stomach and occasionally in the small intestine.^[1]^ They are usually classified into phytobezoars (plant material), trichobezoars (hair), lactobezoars (milk), and pharmacobezoars (medications) according to their composition.^[2]^ Trichobezoars are the accumulation and impaction of ingested hairs in the gastrointestinal tract.^[1,2]^ They often result from continuous and prolonged habitual ingestion of human hair as well as fur from dolls, animals, and blankets. Trichobezoars are usually retained in the stomach. Rapunzel syndrome is a rare type of gastric trichobezoar with a long tail extending into the distal intestine causing intestinal obstruction.^[3,4]^

Trichobezoars are usually encountered in young females with emotional distress, mental retardation, and psychological disorders including depression, anxiety, pica, and trichotillomania.^[5]^

Symptoms of gastric trichobezoar are often vague, insidious, and nonspecific including nausea, vomiting, abdominal pain, early satiety, loss of appetite, and weight loss. They can be complicated by gastric mucosal erosion, ulceration, bleeding, perforation, gastric outlet obstruction, acute pancreatitis, obstructive jaundice, protein-losing enteropathy, and hypochromic anemia.^[6–8]^

Plain abdominal radiography and upper gastrointestinal series with contrast may reveal a mottled heterogeneous mass that may be mistaken for a food-filled stomach. Abdominal CT scan demonstrates a characteristic well-defined mottled intraluminal mass containing air bubbles. However, upper gastrointestinal endoscopy is the gold standard for diagnosis because it allows direct visualization of the bezoar, which directs further management.

Gastric trichobezoar can be removed endoscopically or surgically. A small-sized trichobezoar can be retrieved endoscopically by using a basket or a snare.^[6]^ Wang et al reported that a 14-year-old girl with a huge gastric trichobezoar was successfully treated by endoscopic retrieval using a polypectomy snare and an electrosurgical knife.^[9]^ However, gastric trichobezoar often requires surgical removal because the tightly woven hairs appear not amenable to chemical softening or endoscopic removal.

With the advent of laparoscopic surgery, gastric trichobezoar has been removed by laparoscopic approach.^[10–16]^ Nirasawa et al first reported a 7-year-old girl who underwent laparoscopic gastrotomy and retrieval through the suprapubic mini-laparotomy.^[13]^ Tudor et al also reported 2 cases of gastric trichobezoars that were removed by laparoscopic-assisted technique with wound retractor.^[14]^ Although laparoscopic surgery is advantageous in view of cosmetic results and shorter length of hospital stay, an open approach is still performed in cases of large gastric trichobezoar due to the shorter operative time and less complication rate.^[1,6]^

## 4. Conclusion

Although the recurrence of gastric trichobezoar after surgery is rare, few recurrent cases were reported in the literature as in case 1 of ours.^[17–19]^ Therefore, psychiatric consultation and regular follow-up after treatment should be considered in the children and their parents to prevent the recurrence of gastric trichobezoar.

## Author contributions

**Conceptualization:** Hyung Jun Kwon, Jinyoung Park.

**Formal analysis:** Hyung Jun Kwon, Jinyoung Park.

**Writing – original draft:** Hyung Jun Kwon, Jinyoung Park.

**Writing – review & editing:** Jinyoung Park.
